# The impact of lateral ankle ligament injuries on ankle stability and talar cartilage stress: a finite element analysis of combined injury mechanisms

**DOI:** 10.3389/fbioe.2025.1697096

**Published:** 2025-10-21

**Authors:** Shuai Ji, Liang Sun, Qian Wang, Hongfei Qi, Bo Wu, Bing Du, Chengcheng Zhang, Kun Zhang, Zhong Li, Ming Li, Yao Lu

**Affiliations:** Department of Orthopaedic Surgery, Honghui Hospital, Xi’an Jiaotong University, Xi’an, Shaanxi, China

**Keywords:** ankle lateral collateral ligament, finite element, anterior talofibular ligament, calcaneofibular ligament, biomechanics

## Abstract

**Objective:**

To investigate the differential biomechanical effects of injuries to the superior and inferior fascicles of the anterior talofibular ligament (ATFL) and the calcaneofibular ligament (CFL) on ankle stability and talar cartilage stress, providing a basis for the Fascicle-specific diagnosis and treatment of chronic ankle instability.

**Methods:**

A finite element model incorporating bones, articular cartilage, and nine sets of ligament spring elements was created based on CT data from the ankle of a 26-year-old male volunteer. Six conditions were simulated: 1. intact model; 2. superior ATFL Fascicle injury; 3. inferior ATFL Fascicle injury; 4. complete ATFL injury (superior + inferior Fascicles); 5. CFL injury; and 6. combined ATFL + CFL injury. Anterior drawer test (100 N anterior traction), varus stress test (1.7 N·m torque), and single-leg standing test (600 N axial load) were performed to measure talar displacement and cartilage stress distribution.

**Results:**

1. In the anterior drawer test, talar displacement was greater in superior ATFL Fascicle injuries than in inferior ATFL Fascicle and CFL injuries. 2. In the inversion test, talar displacement was greater in CFL injuries than in superior, inferior, or complete ATFL injuries. 3. In the single-leg standing test, all five injury models altered the contact stress distribution on the talar cartilage compared to the intact model, shifting the peak stress from the anterolateral dome to the anteromedial dome and increasing pressure on the anteromedial talar dome. The peak stress magnitudes were ranked as follows: ATFL + CFL injury > complete ATFL injury > superior ATFL Fascicle injury > inferior ATFL Fascicle injury > CFL injury. 4. The combined ATFL + CFL injury model showed significantly greater displacement and peak stress than the other four injury models.

**Conclusion:**

The superior Fascicle of the ATFL is the primary restraint for anterior stability; the CFL dominates rotational stability; combined injuries trigger a biomechanical cascade failure; lateral ankle ligament injuries lead to increased contact stress on the anteromedial talar cartilage, contributing to the development of arthritis.

## 1 Introduction

Lateral ankle ligament injuries represent one of the most common orthopedic injuries in sports medicine, particularly prevalent among athletic populations. The anterior talofibular ligament (ATFL) and the calcaneofibular ligament (CFL) form the core of the lateral ligamentous complex and play crucial roles in maintaining ankle stability: the ATFL acts as the primary restraint to anterior translation of the talus, while the CFL, spanning both the ankle and subtalar joints, primarily controls inversion and rotational stability (Chen et al., 2025; Maduka, Jakusonoka, Maduka and Yusuf, 2023). Typical injury patterns include isolated ATFL tears or combined ATFL-CFL injuries, which frequently progress to chronic ankle instability (CAI) and predispose individuals to subsequent talar cartilage degeneration and osteoarthritis (OA).

Despite significant advancements in clinical diagnosis and repair techniques, substantial knowledge gaps persist regarding the precise biomechanical mechanisms of lateral ligament injuries, such as the interactive effects of Fascicle-specific ATFL injuries and CFL deficiencies. Traditional anatomical studies and clinical observations are limited in their ability to quantify post-injury alterations in stress distribution. Finite element analysis (FEA), which constructs three-dimensional models to simulate biomechanical responses *in silico*, offers a powerful tool for systematically investigating the impact of different injury conditions (e.g., Fascicle-specific ATFL tears or CFL deficiency) on joint stability and cartilage contact mechanics (Larkins et al., 2021; Mercan et al., 2024). However, previous FEA studies have often simplified the ATFL as a single, homogeneous structure. A growing body of evidence now confirms that the ATFL consists of superior and inferior Fascicles with distinct biomechanical functions (Kakegawa et al., 2019; Ruzik et al., 2025). Concurrently, the dominant role of the CFL in rotational stability has not been sufficiently quantified using FEA.

Addressing these limitations, this study innovatively employs a finite element modeling approach to specifically investigate the combined scenarios of ATFL Fascicle differentiation (superior vs. inferior) and CFL injury. By systematically simulating a sequence of ligament injuries, we aim to quantitatively analyze their effects on anterior stability, inversion stability, and talar cartilage stress distribution. This research seeks not only to elucidate the biomechanical mechanisms of Fascicle-specific injuries but also to provide a theoretical foundation for Fascicle-specific diagnosis and treatment, thereby facilitating the optimization of precise repair strategies. The core novelty of this study lies in the first application of an FEA model incorporating subdivided ATFL Fascicles combined with a multi-variable model including CFL injury, aiming to bridge current knowledge gaps and inform clinical practice.

## 2 Methods

### 2.1 Data collection and model establishment

A 26-year-old healthy male volunteer (height: 175 cm, weight: 70 kg) was selected for this study. X-ray examination confirmed the absence of ankle pathologies or bone abnormalities, and the participant had no relevant medical history. Thin-slice computed tomography (CT) scanning of the ankle was performed using a SOMATOM Definition AS + scanner (Siemens, Germany) with a voltage of 120 kV, current of 150 mA, and slice thickness of 0.6 mm. The tomographic images were saved in Dicom format. This study was approved by the Medical Ethics Committee of the Honghui Hospital Xi’an Jiaotong University (Approval No.: 202407006). The volunteer provided informed consent and signed the consent form.

**FIGURE 1:**
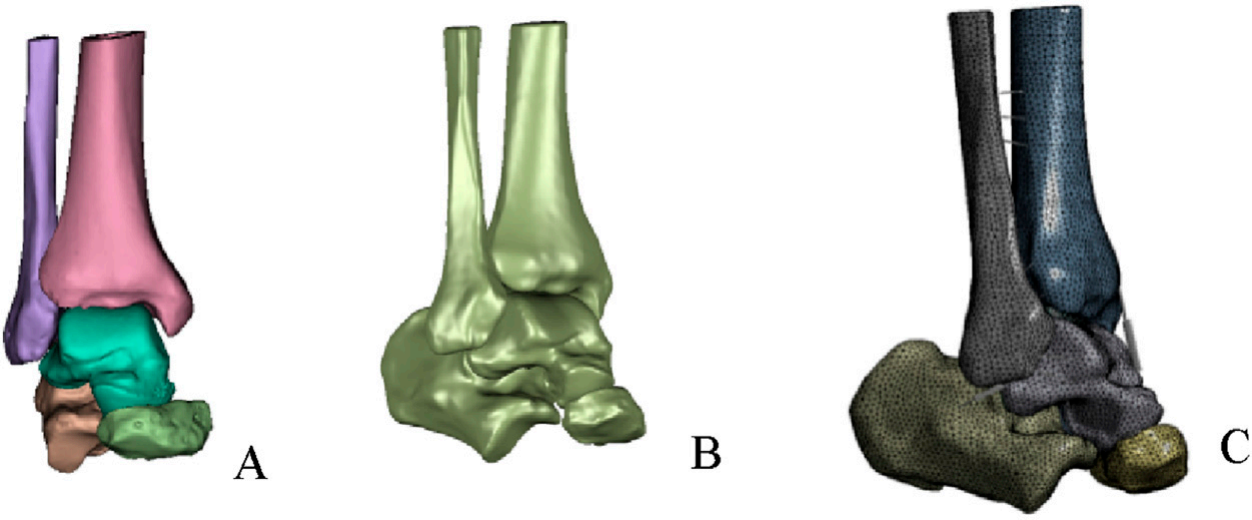
Establishment of finite element model. **(A)** Preliminary ankle joint model. **(B)** Overall fitted surface model. **(C)** Complete ankle joint mode.

A preliminary three-dimensional model of the ankle, comprising five required bones (tibia, fibula, talus, calcaneus, and navicular), was constructed in Mimics 21.0 (Materialise, Belgium) using commands such as threshold segmentation and region growing (Figure 1A). The model was exported in STL format and imported into Geomagic 2021 (Raindrop Geomagic, USA) for further processing, including remeshing, smoothing, noise reduction, removal of spikes, and feature elimination. Finally, a solid model was generated using the surface fitting command (Figure 1B) and stored in STEP format. The model was then imported into SolidWorks 2021 (Dassault Systèmes, France), where the tibia, fibula, talus, and other bones were assembled into anatomical positions based on origin matching. Subsequently, the model was saved in part format, articular cartilage was created, and the analysis model was obtained through Boolean operations and interference checking.

### 2.2 Finite element analysis of the model

The model was imported into Ansys 2021 (ANSYS Inc., USA). Material parameters for the ankle bones and articular cartilage were assigned, with the assumption that all materials were continuous, isotropic, homogeneous, and linearly elastic. Specific material properties are provided in Table 1. We utilized spring elements to model the ligaments and tendons surrounding the ankle joint. Both the superior and inferior fascicles of the anterior talofibular ligament (ATFL) originate from the fibular head and attach anteriorly to the talar neck. The origin and insertion points of the superior fascicle are located superior to those of the inferior fascicle (Hong et al., 2023). The lengths of both fascicles were determined based on previously reported mean values from the literature (Vega et al., 2020). Furthermore, the total stiffness of the intact ATFL, as documented in previous studies, was proportionally allocated to the superior and inferior fascicles according to their estimated cross-sectional areas (Hong et al., 2023; Hoefnagels, Waites, Wing, Belkoff and Swierstra, 2007) (Table 2). To preserve fine structural features and improve meshing efficiency, both bony structures and articular cartilage were discretized using second-order tetrahedral elements. The interfaces between bone and cartilage were defined as “bonded”, while cartilage-to-cartilage contact was set as “frictional” with a coefficient of friction of 0.1 (Figure 1C).

**TABLE 1:** Material parameters of bones and cartilage in the ankle joint (Wang et al., 2018).

Material name	Elastic modulus (MPa)	Poisson’s ratio
Tibia	7300	0.3
Fibula	7300	0.3
Talus	7300	0.3
Calcaneus	7300	0.3
Navicular	7300	0.3
Articular Cartilage	10	0.4

**TABLE 2:** Spring stiffness values of ligaments and tendons in the ankle joint **(** Hoefnagels et al., 2007).

Ligament	Spring stiffness (N/mm)
Superior Anterior talofibular ligament	108.6
Inferior Anterior talofibular ligament	33.2
Calcaneofibular ligament (CFL)	80.0
Posterior talofibular ligament	82.0
Anterior inferior tibiofibular ligament	78.0
Posterior inferior tibiofibular ligament	101.0
Anterior tibiotalar ligament	122.6
Tibiocalcaneal ligament	80.0
Posterior tibiotalar ligament	60.0
Tibionavicular ligament	80.0
Interosseous membrane	400.0

Six finite element models of the ankle were established: 1. Intact model; 2. Superior ATFL Fascicle injury model: spring elements representing the superior Fascicle of the ATFL were deleted; 3. Inferior ATFL Fascicle injury model: spring elements representing the inferior Fascicle of the ATFL were deleted; 4. Complete ATFL injury model: spring elements representing both the superior and inferior Fascicles of the ATFL were deleted; 5. CFL injury model: spring elements representing the CFL were deleted; 6. Combined ATFL + CFL injury model: spring elements representing both the complete ATFL and the CFL were deleted.

**FIGURE 2:**
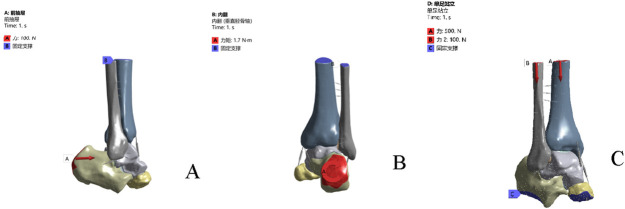
Boundary conditions and loads under different operating conditions. **(A)** anterior drawer test simulation. **(B)** inversion test simulation. **(C)** the single-leg standing test.

Boundary and loading conditions were applied as follows: ① Anterior drawer test: all degrees of freedom at the proximal sections of the tibia and fibula were constrained, and a 100 N anterior traction force was applied to the head of the calcaneus (Figure 2A); ② Inversion test: all degrees of freedom at the proximal sections of the tibia and fibula were constrained, and a 1.7 N·m internal rotation torque was applied along the lateral longitudinal axis of the calcaneus (Figure 2B); ③ Single-leg static standing test: based on previous literature, the load distribution ratio between the tibia and fibula was set at 5:1. The bottom of the calcaneus and the anterior aspect of the navicular were fixed. Vertical downward loads of 500 N and 100 N were applied to the proximal sections of the tibia and fibula, respectively (Figure 2C).

### 2.3 Data analysis

The following outcome measures were evaluated:1. Peak displacement and distribution nephograms of the talus during the anterior drawer test; 2. Peak displacement and distribution nephograms of the talus during the inversion test; 3. Contact stress distribution and the location of peak stress on the talar dome during the single-leg standing test.

## 3 Results

### 3.1 Validation of the finite element model

The established finite element (FE) model of the intact ankle joint comprised a total of 384,803 nodes and 262,158 elements. The model’s validity was tested by simulating the anterior drawer test and the inversion stress test to assess ankle stability. The results demonstrated that the average talar displacement was approximately 1 mm in both simulations: 1.18 mm (range: 0.39–2.06 mm) for the anterior drawer test and 0.89 mm (range: 0.67–1.14 mm) for the inversion test. Furthermore, during the simulated single-leg standing test, the peak stress on the talar dome cartilage was 2.52 MPa, located in the anterolateral region. This finding is consistent with the peak cartilage stress (2.74 MPa) and its location (anterolateral) reported in cadaveric experiments by Anderson et al., further validating the effectiveness of the present model.

### 3.2 Anterior drawer test

**FIGURE 3:**
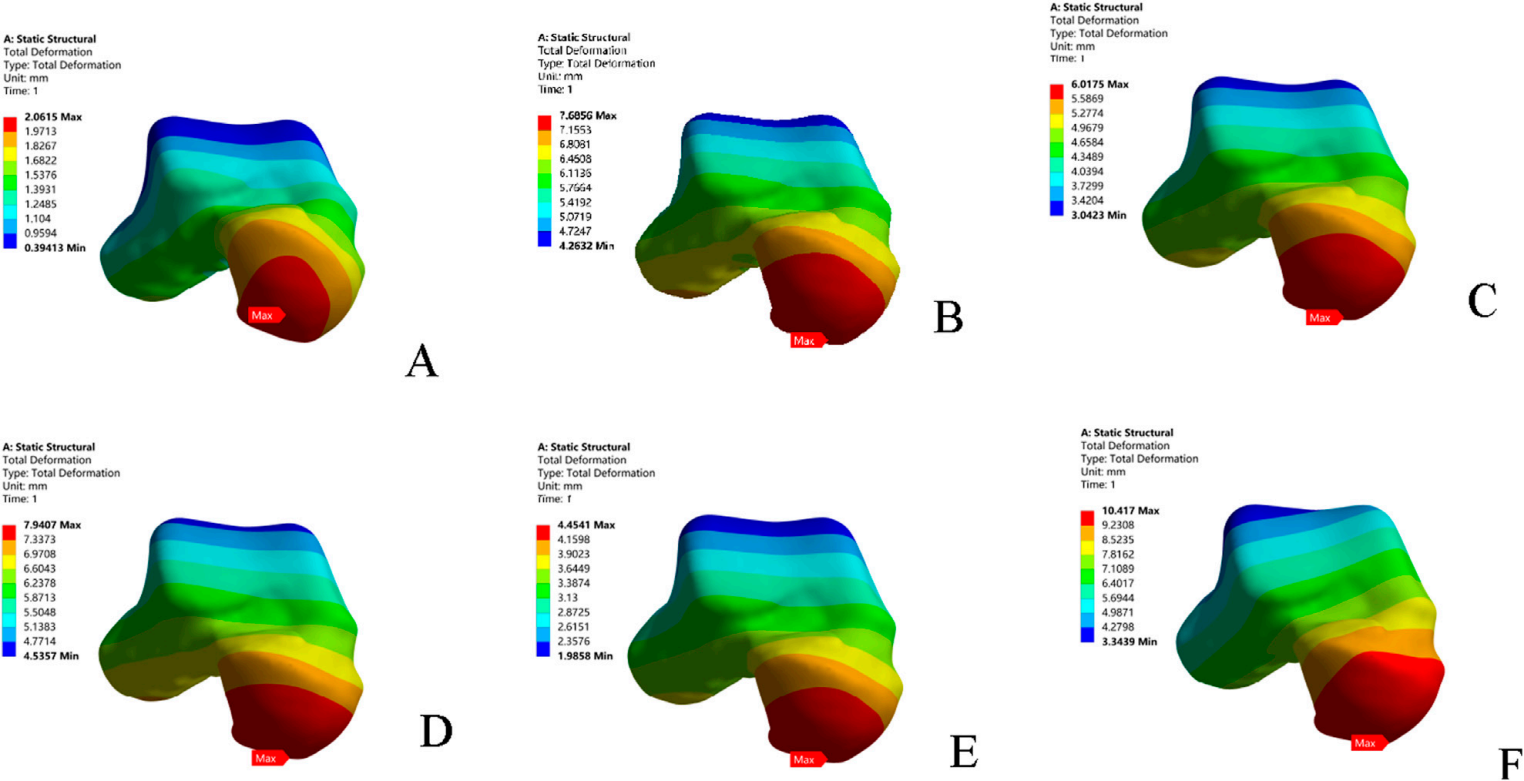
Displacement nephograms of the six models under anterior drawer loading. **(A)** Intact model; **(B)** Superior ATFL injury model; **(C)** Inferior ATFL injury model; **(D)** ATFL injury model; **(E)** CFL injury model; **(F)** ATFL + CFL injury model. ATFL: anterior talofibular ligament; CFL: calcaneofibular ligament.

Under anterior drawer loading, the peak and average talar displacements for the superior fascicle of the ATFL were greater than those for its inferior fascicle. The talar displacement observed in the CFL-deficient model was lower than that in models with injury to the superior fascicle, inferior fascicle, or the entire ATFL. The model with combined ATFL (complete) and CFL injury exhibited significantly higher peak and average talar displacements than the other four injury models. Compared to the intact model, the location of the peak talar displacement in the injury models shifted progressively from the anterolateral aspect of the talar head towards the medial side (Table 3; Figure 3)

**TABLE 3:** Talar displacement results under anterior drawer test loading.

Model	Talar displacement (mm)			Increase vs. Intact (%)
	Min	Max	Average	
Intact Model	0.39	2.06	1.18	—
Superior ATFL injury	4.26	7.69	5.86	397%
Inferior ATFL injury	3.04	6.02	4.41	274%
ATFL Injury	4.54	7.94	6.08	415%
CFL Injury	1.99	4.45	3.12	164%
ATFL + CFL injury	3.34	10.41	6.80	478%

### 3.3 Varus stress test

**FIGURE 4:**
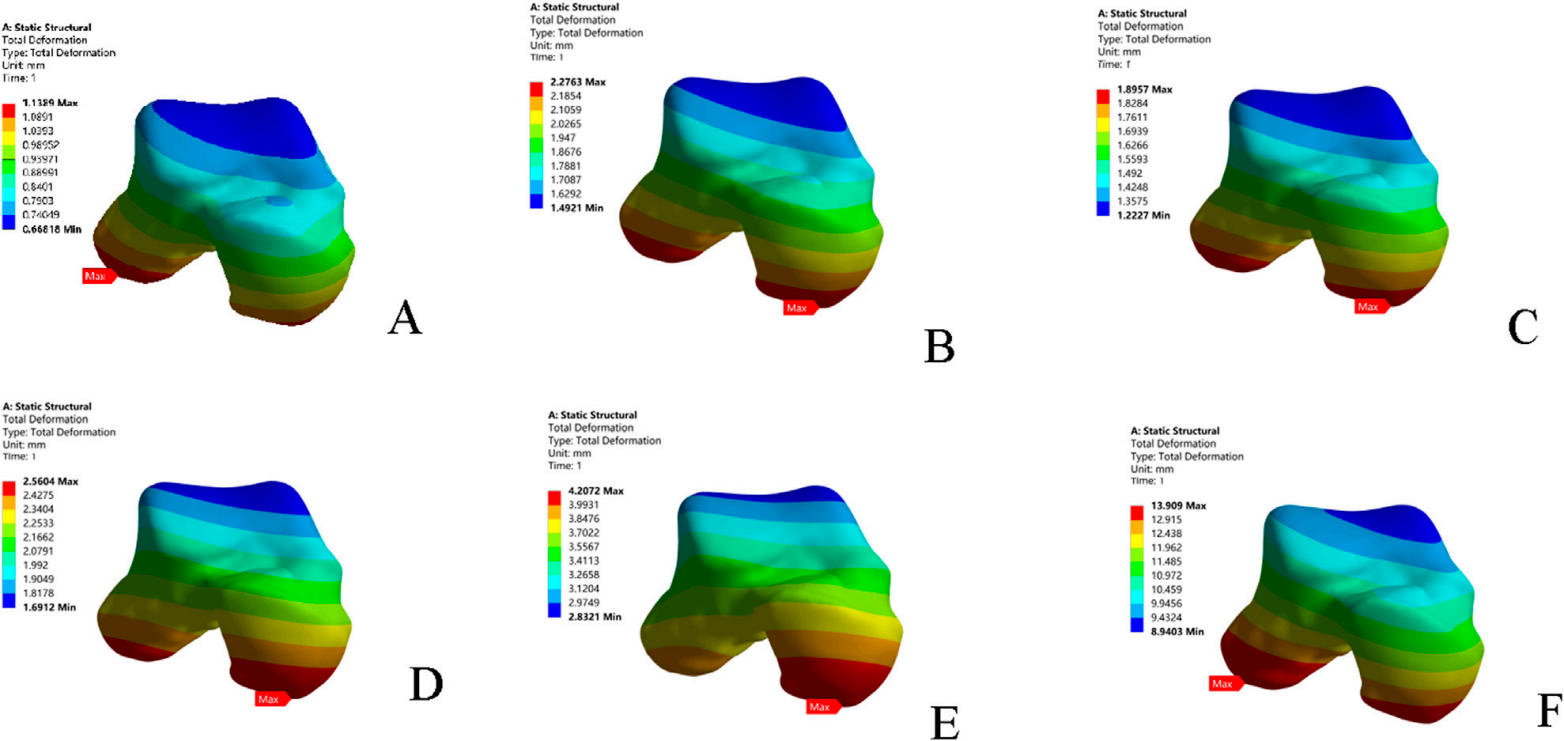
Displacement nephograms of the six models under inversion loading. **(A)** Intact model; **(B)** Superior ATFL injury model; **(C)** Inferior ATFL injury model; **(D)** ATFL injury model; **(E)** CFL injury model; **(F)** ATFL + CFL injury model. ATFL: anterior talofibular ligament; CFL: calcaneofibular ligament.

In the inversion test, the CFL-deficient model resulted in higher peak and average talar displacements than models with injury to the individual fascicles (superior or inferior) or the entire ATFL. The superior fascicle of the ATFL contributed to greater talar displacement (both peak and average) than the inferior fascicle. The combined ATFL (complete) and CFL injury model showed significantly greater talar displacement than the other four injury models. The location of peak talar displacement also changed among the models: it was located at the lateral process of the talus in the intact model; shifted to the talar head following injury to any ATFL fascicle; and migrated back to the lateral process in the combined injury model. The overall directional trend of this shift was anteromedial (Table 4; Figure 4).

**TABLE 4:** Talar displacement results under inversion test loadin**g**.

Model	Talar displacement (mm)			Increase vs. Intact (%)
	Min	Max	Average	
Intact Model	0.67	1.14	0.89	-
Superior ATFL injury	1.49	2.28	1.88	111%
Inferior ATFL injury	1.22	1.90	1.55	74%
ATFL Injury	1.69	2.56	2.10	136%
CFL Injury	2.83	4.21	3.47	290%
ATFL + CFL injury	8.94	13.91	11.30	1166%

### 3.4 Single-leg standing test: Peak cartilage stress and evolution

**FIGURE 5:**
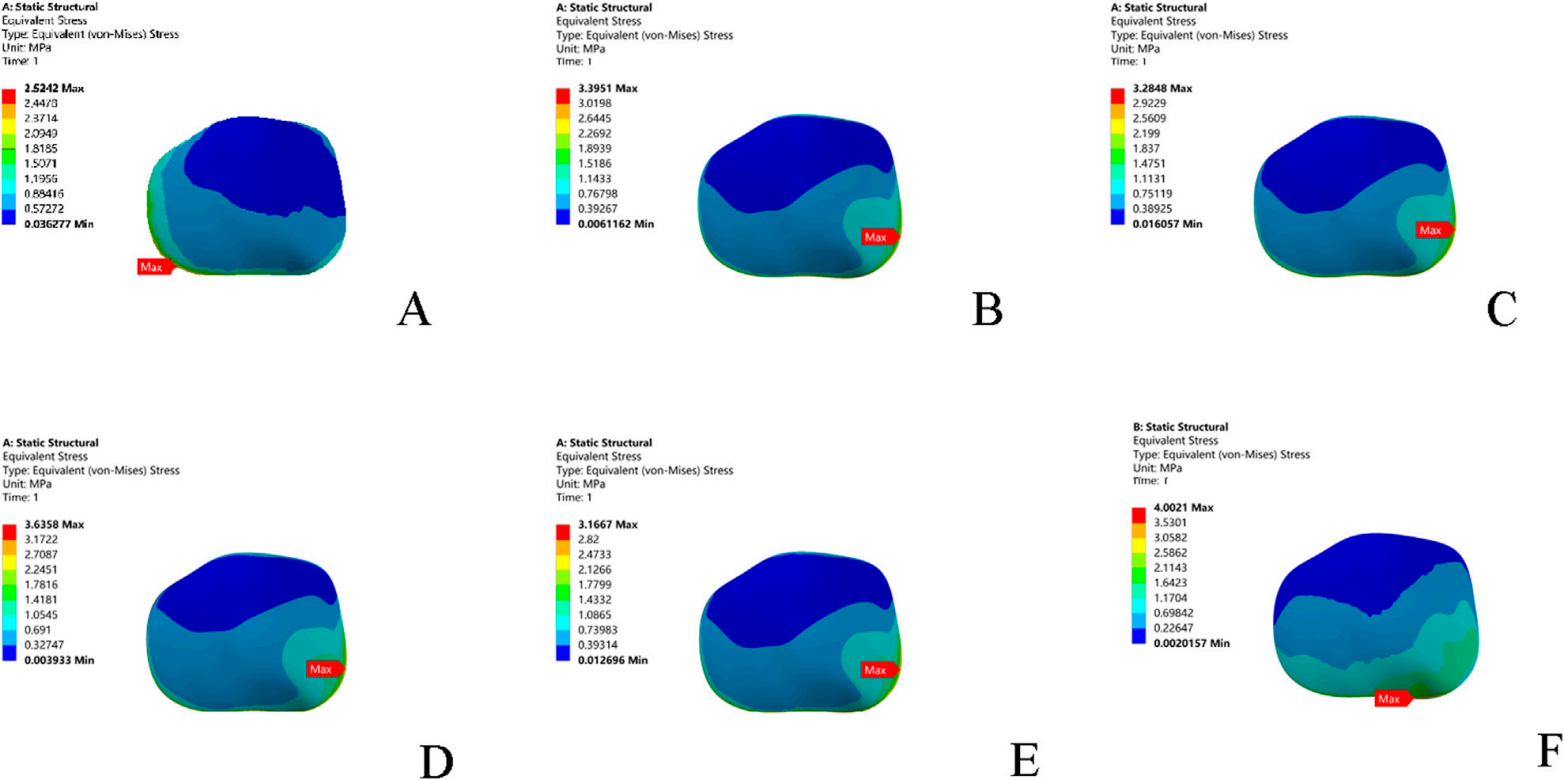
Stress nephograms (von Mises stress) of the six models under single-leg standing loading. **(A)** Intact model; **(B)** Superior ATFL injury model; **(C)** Inferior ATFL injury model; **(D)** Complete ATFL injury model; **(E)** CFL injury model; **(F)** Complete ATFL + CFL injury model. ATFL: anterior talofibular ligament; CFL: calcaneofibular ligament.

Compared to the intact model, all five injury models altered the contact stress distribution on the talar cartilage. The peak stress location shifted from the anterolateral region of the talar dome to the anteromedial region, leading to increased pressure on the anteromedial aspect of the talar dome. In terms of the magnitude of the peak von Mises stress: the combined ATFL + CFL injury model exhibited a higher stress peak than the other four injury models; injury to the superior fascicle of the ATFL resulted in a higher peak stress than injury to the inferior fascicle; and the CFL-deficient model showed a lower peak stress than the other models (Table 5; Figure 5).

**TABLE 5:** Talar stress results under single-leg standing test loadin**g**.

Model	Peak stress (MPa)	Stress concentration region	Increase vs. Intact
Intact Model	2.52	Anterolateral	-
Superior ATFL injury	3.40	Anteromedial	34.9%
Inferior ATFL injury	3.28	Anteromedial	30.2%
ATFL Injury	3.64	Anteromedial	44.4%
CFL Injury	3.17	Anteromedial	25.8%
ATFL + CFL injury	4.00	Extensive Anteromedial	58.7%

## 4 Discussion

Ankle sprain is one of the most common injuries in sports medicine and is also considered the most frequent cause of osteochondral lesions of the talus. The articular surface of the talus is wider anteriorly and narrower posteriorly, making the ankle joint more lax in plantarflexion than in dorsiflexion. Additionally, the lateral collateral ligament complex is weaker than the medial deltoid ligament, the invertor muscles are more numerous and exert a greater force than the evertors, and the lateral malleolus is positioned lower than the medial malleolus. Consequently, the ankle is particularly susceptible to sprains in the inverted and plantarflexed position, often resulting in injury to the lateral ligament complex and frequently leading to chronic ankle instability. Previous studies have indicated that 80% of these injuries involve isolated tears of the anterior talofibular ligament (ATFL), while 20% involve combined ruptures of the ATFL and the calcaneofibular ligament (CFL) (Vega et al., 2020). However, a growing body of anatomical evidence now suggests that the ATFL consists of superior and inferior fascicles, which play distinct roles in maintaining ankle stability. This study employed finite element analysis (FEA) to systematically investigate the impact of different injury patterns of the lateral ankle ligaments on ankle stability and talar cartilage contact stress. Its innovation lies in the subdivision of the ATFL into superior and inferior fascicles, and the examination of multiple injury scenarios incorporating CFL damage, thereby revealing the biomechanical mechanisms specific to fascicle-specific injuries.

### 4.1 Differential biomechanical effects of fascicle-specific ATFL injuries

The anatomical variation of the ATFL is complex. While most prior studies have treated it as a single ligament, an increasing number of recent cadaveric studies have identified that it typically comprises two fascicles (Cordier et al., 2021). In this finite element model, the ATFL was separated into a superior fascicle and an inferior fascicle to simulate the biomechanical response following injury to each. The results demonstrate significant differences in ankle stability impairment depending on the injured fascicle, providing a basis for the precise clinical identification of micro-instability.

#### 4.1.1 Superior fascicle injury: the biomechanical basis of micro-instability

In this study, it is confirmed for the first time in the finite element model that ATFL superior Fascicle injury has a significant effect on talus forward movement and internal rotation, especially on the forward stability, which is consistent with the results of a robot study (Dalmau-Pastor et al., 2023). We observed that the defect of the superior Fascicle alone led to an average increase of 39.7% (5.86 mm) in the anterior drawer test, which was more significant than the injury of the lower Fascicle (increase of 27.4%). This result is consistent with cadaveric research: the superior Fascicle of fibers mainly limits the forward displacement under plantar flexion, and its injury can lead to anteromedial stress concentration (Kakegawa et al., 2019; Ruzik, Czech et al., 2025). The analysis of the displacement nephogram further revealed that the peak displacement after the superior Fascicle defect moved from the anterolateral side to the anteromedial area, forming a characteristic “anteromedial displacement concentration sign”. This change stems from the intra-articular nature of the superior fasciculus. Although the injury did not cause the relaxation of the macro ligament, it triggered a slight forward movement and internal rotation (Vega et al., 2020). This mechanism may explain the lack of objective signs in 30%–40% patients with ankle sprain, which is consistent with the concept of “ankle slight instability” proposed by Vega et al. (2021). It is worth noting that the peak displacement migration may lead to abnormal load on the anterior medial articular surface of the talus, which becomes a potential cause of cartilage degeneration in the long term.

At present, more and more researches divide chronic ankle instability into classic type (combined rupture of ATFL and calcaneal ligament (CFL)) and slight instability type (injury of superior Fascicle of ATFL alone). Slight instability is a new concept in ankle joint. The pathological mechanism proposed at present is that after ankle varus injury, the superior fascicle of ATFL is the first torn ligament, resulting in slight instability of ankle joint (Vega et al., 2017; Vega et al., 2016). The superior fascicle of ATFL is an intra-articular structure, which is extremely difficult to recover after injury. If the injury force continues, the lower fascicle of ATFL and CFL will be injured next, and the patient will eventually have mechanical ankle instability (Vega et al., 2020). Traditionally, we have used clinical evaluation tools (such as front drawer test and talus varus stress test) to judge ankle ligament injury, so as to evaluate the integrity of ATFL and CFL, but the simple ATFL superior fascicle injury may not be detected by this traditional examination. In addition, most hospitals usually report the damage of the whole ATFL as a single structure in imaging research, such as magnetic resonance imaging and ultrasonic examination. All these suggest that we should re-establish a complete diagnosis and treatment system of fasciculation injury to evaluate ankle joint injury in the clinic.

#### 4.1.2 Inferior fascicle injury: a secondary contributor to anterior translation and rotatio

The anatomical course of the inferior Fascicle (originating from the anterior border of the distal fibula and inserting obliquely on the talar neck) directs its mechanical vector more towards restricting anterior translation. In comparison, injury to the inferior Fascicle had a relatively smaller impact on anterior displacement (274% increase vs. 397% for the superior Fascicle). In the inversion test simulation, the increase in inversion displacement caused by inferior Fascicle injury (74%) was significantly lower than that caused by CFL injury (290%), supporting its secondary role in rotational restraint. However, its potential contribution should not be overlooked in clinical practice and must be assessed in the context of the overall ligamentous status.

### 4.2 The unique biomechanical phenotype of CFL injury

The anatomical position of the CFL, spanning both the ankle and subtalar joints, with its fibers oriented perpendicular to the axis of the ankle joint, establishes it as the primary restraint against rotation under inversion stress (Hunt et al., 2019). Our inversion test simulation confirmed this, demonstrating that isolated CFL injury resulted in a 290% increase (3.47 mm) in talar displacement, substantially higher than the 136% increase caused by complete ATFL injury. Displacement nephrograms revealed that CFL deficiency caused the peak displacement to shift from the lateral process of the talus anteromedially, an effect that underscores the role of the CFL as the primary rotational restraint. This result biomechanically emphasizes the necessity of prioritizing CFL assessment in the clinical diagnosis of inversion instability. In the anterior drawer test, the displacement increase for CFL injury (164%) was far lower than for superior ATFL injury (397%), supporting the classic theory of its role as a secondary anterior restraint. It is noteworthy that although CFL injury did not significantly increase the magnitude of anterior translation, it still altered the trajectory of talar motion (medial shift of the peak displacement point), suggesting it indirectly influences anterior stability by modulating talar rotation (Li et al., 2019).

### 4.3 The cascading amplification effect of combined injuries

The model combining complete ATFL and CFL injuries revealed that multi-ligament deficiencies trigger a synergistic amplification effect, severely compromising joint stability and cartilage integrity. In the anterior drawer test, talar anterior translation in the combined injury model reached 6.80 mm (a 478% increase compared to the intact model), while displacement in the inversion test surged to 11.30 mm (a 1166% increase), significantly exceeding values in the isolated injury models. We postulate the occurrence of a complex three-dimensional displacement coupling phenomenon. 1. Antero-medio-inferior gliding tendency: The peak displacement point migrated to the medial body of the talus in the anterior drawer test and shifted towards the talocalcaneal articular surface in the inversion test. 2. Loss of tibiotalar congruence: Nephrograms indicated the talus exhibited a compound motion of anterior translation coupled with internal rotation, and the articular contact zone shifted anteriorly. 3. Displacement magnitude multiplier effect: The increase in displacement for the combined injury was significantly greater than the sum of the isolated injuries (e.g., 1166% increase in inversion displacement vs. the theoretical sum of individual ATFL and CFL increases). This cascading effect is consistent with the theory of rotational ankle instability: when both the ATFL and CFL are deficient, the talus loses its bi-directional restraint, resulting in multi-plane uncontrolled displacement (Boey et al., 2019). The migration path of the peak displacement point suggests a staged biomechanical failure: anterior translation dominates in isolated ATFL injuries, which progresses to a compound motion of anterior translation-inversion-internal rotation in combined injuries.

### 4.4 Clinical implications of altered cartilage stress distribution

Simulations under the single-leg standing condition using the finite element model revealed systematic changes in cartilage stress distribution. In the intact ankle, the peak stress was located in the anterolateral region of the talus (approximately 2.52 MPa). Following an injury to the superior Fascicle of the ATFL, the peak stress shifted to the anteromedial region (approximately 3.40 MPa). In scenarios of combined ligament injuries (e.g., ATFL-CFL), the peak stress further increased to approximately 4.00 MPa and exhibited a diffuse distribution pattern. The mechanism underlying this stress shift can be explained by the fact that Injuries to the lateral collateral ligament lead to reduced ankle stability, causing the talus to exhibit a kinematic tendency of anterior translation and internal rotation during weight-bearing. This complex motion alters the contact characteristics of the tibiotalar joint, shifting the contact zone anteromedially. This finding is supported by Caputo et al. (2009), who also suggested that increased internal rotation and anterior translation may shift the contact point anteromedially in cases of ATFL injury. Furthermore, related biomechanical experiments have validated similar changes, showing significant abnormalities in radiographic parameters (e.g., T-value) in patients with lateral ankle ligament injuries, which are associated with an increased risk of cartilage degeneration (Hu et al., 2018). Secondly, this study quantitatively confirmed a positive correlation between the severity of ligament injury and the degree of abnormal stress distribution. This supports the theoretical framework proposed by Vega et al. (2020), which describes a cascading pathway from micro-instability to mechanical instability and ultimately to arthritis. Additionally, while acute cartilage injuries have the potential to heal clinically (Blom et al., 2021), chronic instability resulting from lateral ankle ligament injuries causes recurrent joint trauma, impairing the cartilage’s healing capacity. More severe osteochondral lesions of the talus can further disrupt joint congruence, exacerbating ankle instability and creating a vicious cycle. This may explain the challenges in treating ankle osteoarthritis (OA). The clinical implications of these findings include: 1) Early-stage finite element analysis (FEA) simulations may help predict the risk of osteoarthritis (OA) onset, enabling earlier detection, intervention, and treatment; 2) Imaging evidence should be integrated with an assessment of the state of ligamentous Fascicles to improve diagnostic accuracy, particularly by enhancing diagnostic methods for the superior Fascicle of the ATFL; and 3) Ligament injuries drive the progression of cartilage degeneration through biomechanical coupling mechanisms, underscoring that treatment should prioritize the restoration of normal joint kinematics.

### 4.5 Clinical significance of bundle-specific diagnosis and treatment

Based on the findings of this study and evidence from related literature, we propose a novel bundle-specific diagnosis and treatment strategy aimed at optimizing the management of chronic ankle instability. Diagnostic Level: 1. Superior ATFL Fascicle Injuries: Patients with an isolated superior ATFL Fascicle injury often present with negative physical examination findings, leading to a high rate of missed diagnosis. Therefore, for patients with a history of ankle sprain who report subjective ankle instability despite inconclusive physical exams and failed long-term conservative treatment, this study recommends utilizing MRI to assess the integrity of the ligamentous Fascicles (Vega et al., 2021). 2. CFL Injuries: For suspected CFL injuries, stability should be assessed using the inversion stress test, combined with dynamic ultrasonography to evaluate CFL tension. Treatment Decision-Making: Isolated Superior Fascicle Injury: Early arthroscopic repair is recommended for isolated superior Fascicle injuries. Combined Injuries: Combined injuries involving both Fascicles of the ATFL and the CFL necessitate reconstruction of both ATFL Fascicles and the CFL. Current research indicates that combined reconstruction (ATFL-CFL) restores range of motion (ROM) more effectively than isolated reconstruction (Hanada et al., 2022)

### 4.6 Limitations and future directions

This study has several limitations. The model assumed bones and cartilages to be homogeneous, linearly elastic materials, and ligaments were simplified as spring elements, neglecting their nonlinear mechanical behavior. The application of static loading (such as in the anterior drawer test) does not account for the influence of muscle forces during dynamic gait. The finite element model developed in this study did not include other bony structures of the foot, which may potentially affect the stability and stress distribution of the talus and calcaneus. Future work should focus on developing a more comprehensive full-foot model. Additionally, the model was based on data from a single healthy male and lacks validation for females, older adults, or pathological conditions. Moreover, the ATFL was only considered as a two-bundle structure, without accounting for anatomical variations. The morphological dimensions and material parameters of the superior and inferior bundles were simulated based on historical data, which may differ from actual values, and the fiber connections between the inferior bundle and the calcaneofibular ligament (CFL) were not modeled. Future efforts should aim to refine the model by integrating dynamic MRI to validate the time-dependent effects of fascicle-specific ATFL injuries, ultimately translating these insights into clinical applications to improve the diagnosis and treatment of ankle sprains.

## 5 Conclusion

This study, utilizing a refined finite element model, is the first to systematically quantify the differential biomechanical effects of injuries to the superior Fascicle, inferior Fascicle of the ATFL, and the CFL on ankle stability and talar cartilage stress. The key findings are as follows: The superior Fascicle of the ATFL is the primary restraint against anterior talar translation; its injury causes anteromedial stress concentration, forming the biomechanical basis of “ankle micro-instability.” The CFL is the dominant restraint for rotational stability. Combined injuries lead to a synergistic surge in displacement and stress. Injuries to the lateral ankle ligaments mediate the progression of osteoarthritis by increasing stress on the anteromedial talar cartilage. Assessment of occult anterior instability should focus on the integrity of the superior Fascicle; rotational instability requires attention to the state of the CFL, and combined injuries warrant comprehensive reconstruction. The results confirm the distinct biomechanical effects of Fascicle-specific ATFL injuries, emphasize the clinical need to identify micro-instability resulting from superior Fascicle damage, and provide a theoretical basis for the precise reconstruction of combined injuries.

## Data Availability

The raw data supporting the conclusions of this article will be made available by the authors, without undue reservation.
